# Requirements within the Ebola Viral Glycoprotein for Tetherin Antagonism

**DOI:** 10.3390/v7102888

**Published:** 2015-10-26

**Authors:** Nathan H. Vande Burgt, Rachel L. Kaletsky, Paul Bates

**Affiliations:** Department of Microbiology, Perelman School of Medicine at the University of Pennsylvania, 225 Johnson Pavilion, 3610 Hamilton Walk, Philadelphia, PA 19104-6076, USA; nathanv@upenn.edu (N.H.V.B.); kaletsky@princeton.edu (R.L.K.)

**Keywords:** tetherin, BST2, Ebola virus, Ebola, glycoprotein, GP-primed, primed GP, sGP

## Abstract

Tetherin is an interferon-induced, intrinsic cellular response factor that blocks release of numerous viruses, including Ebola virus, from infected cells. As with many viruses targeted by host factors, Ebola virus employs a tetherin antagonist, the viral glycoprotein (EboGP), to counteract restriction and promote virus release. Unlike other tetherin antagonists such as HIV-1 Vpu or KSHV K5, the features within EboGP needed to overcome tetherin are not well characterized. Here, we describe sequences within the EboGP ectodomain and membrane spanning domain (msd) as necessary to relieve tetherin restriction of viral particle budding. Fusing the EboGP msd to a normally secreted form of the glycoprotein effectively promotes Ebola virus particle release. Cellular protein or lipid anchors could not substitute for the EboGP msd. The requirement for the EboGP msd was not specific for filovirus budding, as similar results were seen with HIV particles. Furthermore trafficking of chimeric proteins to budding sites did not correlate with an ability to counter tetherin. Additionally, we find that a glycoprotein construct, which mimics the cathepsin-activated species by proteolytic removal of the EboGP glycan cap and mucin domains, is unable to counteract tetherin. Combining these results suggests an important role for the EboGP glycan cap and msd in tetherin antagonism.

## 1. Introduction

The innate immune system is the first line of defense against viral pathogens. Consequently, mammalian cells employ numerous innate cellular mechanisms to inhibit viral replication and spread. Intrinsic antiviral factors comprise a form of innate immunity that directly limit viral entry, replication or assembly. These factors are often ubiquitously expressed, but can be further induced during viral infection, generally by interferon. Tetherin (also referred to as BST-2, CD317, or HM1.24) is an interferon-inducible host intrinsic antiviral protein that acts at least in part by retaining budded enveloped virions on the cell surface and preventing virion release into the extracellular media [1].

Although discovered as an intrinsic immune factor because of its effect upon HIV-1 replication, the ability of tetherin to disrupt virion budding is not specific for HIV-1. Ebola virus viral particle release is effectively blocked by tetherin [1,2]. Indeed, egress of a range of enveloped viruses including simian lentiviruses, Lassa virus, Kaposi sarcoma-associated herpesvirus, influenza A virus, vesicular stomatitis Indiana virus, Chikungunya virus, and hepatitis C virus are impacted by tetherin expression [1,3,4,5,6]. Tetherin’s ability to inhibit viral particle release from infected cells is dependent upon the protein’s unusual cellular topology which consists of an extracellular coiled coil domain anchored on both ends by a N-terminal transmembrane domain and a C-terminal GPI anchor [7]. During HIV assembly, tetherin is seen to localize to sites of viral budding [8] and block viral egress by forming a physical linkage between the virion and the host cell [9].

For many of the viruses affected by tetherin, antagonists have been identified that impede the anti-viral activity of tetherin. Some of these antagonists, such as HIV-1 Vpu, recognize the transmembrane region of tetherin and modify residues in the cytoplasmic tail of tetherin, resulting in the down-regulation and degradation of tetherin [10]. K5 from KSHV targets residues in the cytoplasmic tail of tetherin for ubiquitination and subsequent proteasomal degradation [11], whereas the envelope proteins of HIV-2 and a subset of SIVs require sequences in the tetherin ectodomain for recognition and surface downregulation [12,13].

Ebola virus is a member of the *Filoviridae* family and a causative agent of outbreaks of hemorrhagic fever in sub-Saharan Africa primarily due to zoonotic transmission of virus from a presumptive natural reservoir in fruit bats [14,15]. Prior to the 2014 epidemic in Western Africa, these outbreaks were infrequent and of limited scope [16]. Ebola virus infection fatality rates are unusually high, ranging from 59%–88%, while disease progression occurs rapidly; on average, patients succumb to infection 10 days after showing symptoms [17,18,19].

Ebola virus infection produces several proteins from the viral glycoprotein (GP) gene. The primary product from the viral GP gene is a 323 residue nonstructural, soluble glycoprotein (sGP) that exists as a homodimer. Polymerase stuttering incorporates an additional nucleotide in a small percentage of the GP transcripts causing a frameshift and production of the full-length, virion associated glycoprotein (EboGP) [20,21]. Due to this method of production, sGP and EboGP share 295 N-terminal residues, including regions within EboGP needed for receptor recognition and cell binding as well as a domain called the glycan cap. EboGP forms trimers and is cleaved in into two subunits, GP_1_ and GP_2_, such that GP_2_ is membrane anchored by a hydrophobic membrane spanning domain (msd) [20].

Structural analysis of EboGP shows that the GP_2_ subunit contains the fusion machinery and forms a stalk that holds GP_1_, the globular receptor-binding region [22]. Within GP_1_ is the glycan cap, a moderately glycosylated region that, together with a heavily glycosylated mucin domain, sits atop the trimeric glycoprotein spike and covers the receptor binding domain of EboGP [22,23]. While EboGP shares the N-terminal 295 residues with sGP, the proteins are markedly different in their structure; EboGP forms trimers, while sGP exists as homodimers [20,24,25].

EboGP has been identified as an inhibitor of intrinsic immunity based upon its ability to act as an antagonist of tetherin [2]. While the mechanism of action for tetherin antagonism by EboGP is poorly understood, tetherin degradation or relocalization from the cell surface is likely not involved [26,27]. Recent reports suggest that EboGP may prevent tetherin from localizing with VP40 [28]. Specific EboGP domains have been implicated in interacting with or counteracting tetherin. Within GP_1_, the mucin domain can be removed without affecting EboGP anti-tetherin activity [2]. Furthermore, FRET analysis of the interaction between EboGP and tetherin has suggested that the GP_2_ subunit appears to interact with tetherin [29]. Similarly recent chimeric protein analysis demonstrated a role for the EboGP msd within GP_2_ in tetherin antagonism [30]. sGP is unable to affect tetherin antiviral function [2].

Here the domains within the Ebolaviral glycoproteins required to antagonize tetherin antiviral activity are further characterized. We define a minimal 320 residue portion of the Ebola glycoprotein ectodomain, containing the receptor binding domain and glycan cap regions of EboGP, that when anchored to the cell surface is sufficient to antagonize tetherin activity. Moreover, there is a specific requirement for the EboGP msd, as anchoring sGP by other cellular msd sequences or by a GPI anchor does not antagonize tetherin activity. Finally, deletion of the glycan cap region by proteolytic processing renders EboGP unable to promote viral budding suggesting that the glycan cap is important for tetherin antagonism.

## 2. Materials and Methods

### 2.1. Cell Lines, Plasmid Vectors and Antibodies

293T cells were grown in DMEM (Invitrogen, Carlsbad, CA, USA) supplemented with 5% fetal bovine serum (Invitrogen) and 2 mM l-Glutamine (Invitrogen). Vectors used to transfect cells were constructed as described below. The vector pcDNA3.1 furin expressing human furin was previously described [31]. To express HIV Gag, psPAX2 was obtained from Addgene (Cambridge, MA, USA). Human tetherin, in the vector pCMV Sport6 Tetherin was obtained from Open Biosystems (Lafayette, CO, USA). An AU1 tagged cytoplasmic tail and transmembrane domain of mouse transferrin receptor one (mtfr1) and the human tetherin ectodomain were combined to generate mtfr1-tetherin in a pCB6 backbone. To express and detect viral protein products, we cloned sequences into the pCAGGS vector and, where specified, appended a C-terminal FLAG, V5, or polyhistidine tag to generate these constructs: pCAGGS VP40 (FLAG tagged), pCAGGS EboGP (V5-His tagged), pCAGGS GP-primed (V5-His tagged), and pCAGGS sGP. EboGP lacking a glycan cap (GP-primed) was generated by replacing EboGP residues 203–206 (VNAT) with a consensus furin cleavage site (RRKR) as previously described [32]. EboGP constructs with amino acid point mutations C670A and C672A were generated both individually and in combination. To generate sGP chimeras, an XbaI restriction site (or XhoI for sGP-TM_(TVA)_) was introduced at the C-terminus of sGP after residue 320, immediately before the furin RVRR cleavage site. Sequences encoding the transmembrane domain from EboGP, human ACE2, the chicken TVA receptor, or a GPI anchored form of the TVA receptor were appended to sGP after the XbaI site. Sequence details of all constructs produced are shown in Supplementary Figure S1. Antibodies used include the mouse IgG2a anti-V5 antibody (Invitrogen 46-0705), rabbit anti-FLAG antibody (Sigma F7425), rabbit polyclonal sera (R12) produced against EboGP [33], and mouse anti-tetherin antibody (Biolegend RS38E, San Diego, CA, USA). HRP-conjugated, Alexa Fluor 488, or Alexa Fluor 647 secondary antibodies against mouse or rabbit Fc were used where indicated.

**Figure 1:**
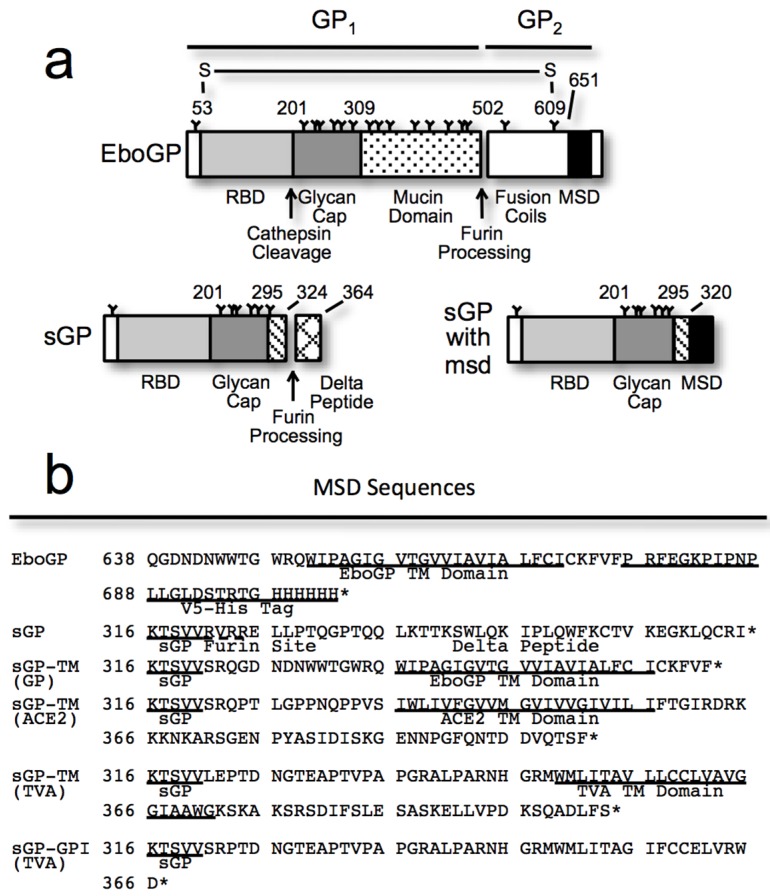

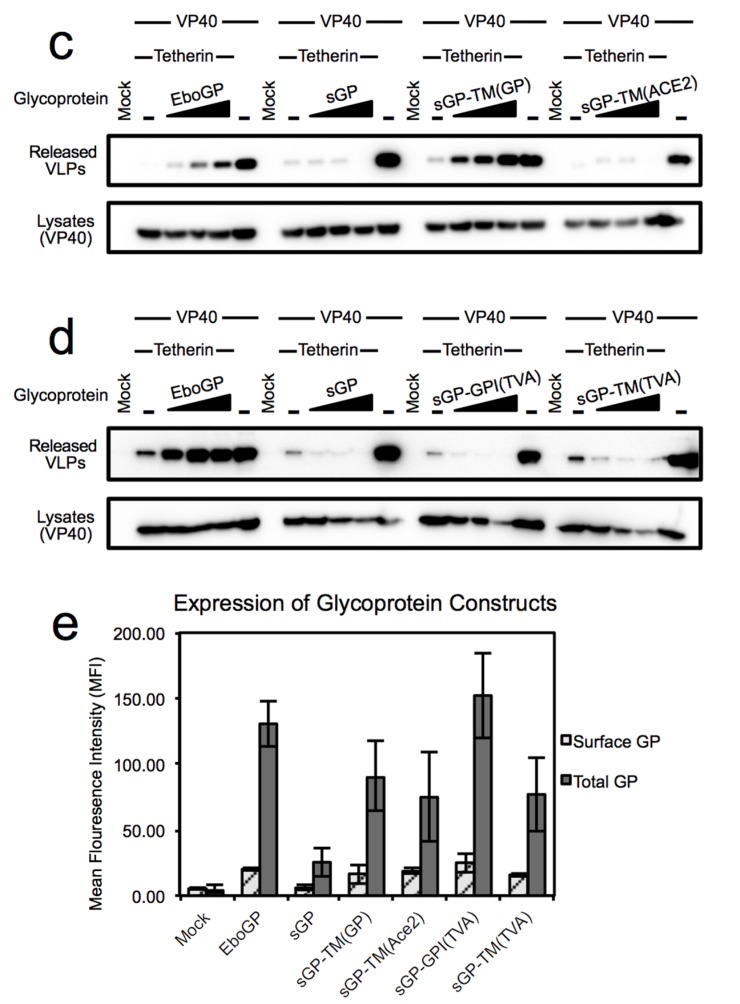
(**a**) A schematic diagram of the constructs used in this study showing the domains of EboGP, sGP, and a chimeric sGP with an appended membrane spanning domain (msd), either a protein transmembrane (TM) domain or a GPI anchor, to the C-terminus; (**b**) Amino acid sequences of the constructs employed, highlighting the C-terminal regions appended to the chimeric sGPs. Where known, the msd sequence is annotated; (**c**) An Ebola virus-like particle (VLP) budding assay comparing the anti-Tetherin activity of EboGP, sGP and chimeric sGPs. 293T cells were transfected with plasmids encoding VP40-FLAG, human Tetherin and varying amounts of the GP constructs as indicated. *Top Panel*: Purified VLPs were analyzed by SDS-PAGE and immunoblot using a FLAG-tag antibody to detect VLPs released into the media. *Bottom Panel*: Cell lysates were analyzed by SDS-PAGE and immunoblot probed with an antibody to FLAG to evaluate VP40 expression in the transfected cells; (**d**) A VLP budding assay comparing the anti-Tetherin activity of EboGP, sGP, and sGP with either a GPI anchor or a proteinaceous msd from TVA. *Top Panel*: Purified VLPs were analyzed by SDS-PAGE and detected by immunoblot and probed with an antibody to the FLAG tag of VP40. *Bottom Panel*: VP40 expression in the 293T cell lysates was confirmed by using an antibody to FLAG in the immunoblot. Results are representative of at least 3 independent experiments; (**e**) A bar chart depicting the relative expression of the GP constructs in 293T cells, as measured by flow cytometry, after staining with a polyclonal antibody (R12) to GP. Surface GP was detected on fixed cells without permeabilization, while for total GP, staining was performed after permeabilization.

### 2.2. Virus-Like Particle (VLP) Budding Assay

293T cells were seeded in a 24-well plate at a density of 1.0 × 10^5^ cells per well. Using Lipofectamine 2000 (Invitrogen) or polyethylenimine (PEI) (PolySciences Inc., Warrington, PA, USA), 293T cells were transfected with plasmids encoding VP40 or psPAX2, tetherin, and filovirus GP or an empty vector. When GP-primed was used, pcDNA3.1 furin was added to all transfected wells. VLPs in the supernatants were harvested at 48 h post-transfection and, after a clarifying spin at 1700 rcf, were purified through a 20% sucrose cushion by centrifugation in a TLA120.1 rotor at 40,000 rpm for 30 min. Concurrently, the cells were lysed in 1% Triton lysis buffer and cleared by centrifugation at 18,000 rcf for 3 min. Cell lysates and purified VLPs were then analyzed by immunoblot.

### 2.3. Immunoprecipitaion Assay

293T cells were seeded in a 6-well plate at a density of 2.2 × 10^5^ cells per well. Using Lipofectamine 2000, 293T cells were transfected with plasmids encoding tetherin, GP, or empty vector. 48 h post-transfection, cells were lysed in a 1% Triton buffer and, after clearing the lysate at 18,000 rcf for 3 min, rocked with Protein A conjugated agarose beads overnight at 4 °C. Concurrently, Protein A conjugated agarose beads were also rocked overnight at 4 °C with antibodies to tetherin (RS38E) or GP (R12). After incubation, the antibody bound agarose beads were washed twice in 1% Triton buffer. The naked agarose beads were cleared from the rocking cell lysates, replaced with antibody bound agarose beads, and rocked overnight at 4 °C. Protein adhering to the antibody bound agarose beads were washed four times in 0.1% Triton buffer and subsequently analyzed by immunoblot.

### 2.4. Immunoblot Analysis

Samples were loaded and run on a 4%–15% Tris-HCl polyacrylamide gel (Bio-Rad, Hercules, CA, USA) and subsequently transferred to a PVDF membrane by electroblotting. Membranes were blocked in 5% non-fat dry milk with Tris-buffered saline (Blotto) and then rocked overnight at 4 °C with a 1:10,000 dilution of antibody. After washing with Blotto, appropriate HRP conjugated secondary antibodies were added to the membranes and rocked for one hour. Membranes were washed in Blotto and Tris-buffered saline with 0.1% Tween-20 and subsequently imaged on a chemiluminescent imager (Fujifilm LAS-1000). Where indicated in experiments, membranes were stripped with Restore Western blot stripping buffer (Thermo Scientific, Waltham, MA, USA) and re-probed with antibodies. All experiments shown are representative of immunoblots repeated at least three times.

### 2.5. Flow Cytometry Analysis

293T cells were seeded at a density of 1.05 × 10^5^ cells per well into a 24-well plate. Cells were transfected using Lipfectamine 2000 with 600 ng of plasmids encoding GP, a chimeric GP or pCAGGS empty vector. 48 h post-transfection, cells were lifted off the plate with 5 mM EDTA in PBS−/− and kept at 4 °C throughout the analysis. Cells were spun at 150× g for 5 min and resuspended in Flow Wash (PBS−/− with 1% FBS and 0.05% NaAz) and probed with an appropriate primary and secondary antibody for 1 h. Cells were washed 2× with Flow Wash, fixed and permeabilized with BD Fix/Perm (BD Biosciences) for 20 min, washed 2× with BD Perm/Wash (BD Biosciences, San Jose, CA, USA), and probed again with appropriate primary and secondary antibody for 1 h. Cells were washed 2× with BD Perm/Wash, resuspended in PBS−/− and analyzed by on a FACS Calibur (BD Biosciences). Post acquisition analysis was performed on FlowJo software (FlowJo LLC, Ashland, OR, USA).

## 3. Results

### 3.1. Requirements within EboGP for Tetherin Antagonism

To define the minimal requirements within the Ebola virus glycoprotein needed to antagonize tetherin function, we employed an Ebola virus-like particle (VLP) budding assay, with a panel of plasmids including full-length EboGP, sGP, an sGP chimera (Figure 1a,b). Previously we found that neither sGP nor secGP, a soluble version of EboGP cleaved at the extracellular base by tumor necrosis factor-converting enzyme (TACE) protease [31], could effectively counteract tetherin [2]. The membrane spanning domain (msd) represents a significant difference between full-length EboGP and secGP; we therefore sought to determine whether the msd was a determinant of anti-tetherin activity. A chimeric glycoprotein was produced by appending the msd from EboGP onto the C-terminus of sGP creating sGP-TM_(GP)_ (Figure 1a,b). Flow cytometry and immunoblot analysis confirmed the expression of the chimeric protein (Figure 1e, Supplementary Figure S2). Analysis of budded Ebola VP40 particles demonstrated that sGP-TM_(GP)_ was able to effectively antagonize tetherin activity by promoting VLP release across a range of sGP-TM_(GP)_ expression levels (Figure 1c). As controls, we confirmed that sGP could not promote virion release, even at the highest levels of sGP expression utilized, while EboGP effectively antagonized tetherin and prompted virion release (Figure 1c). sGP and EboGP expression in transfected 293T cells were verified by flow cytometry (Figure 1e) and by immunoblot of cell lysates and supernatants (Supplementary Figure S2). Cellular lysate expression of sGP appears lower than EboGP because sGP does not contain an msd and thus, is secreted from cells and not retained on the cell surface (Figure 1e). Overall these experiments define a minimal 320 residue portion of the Ebola glycoprotein ectodomain, containing the receptor binding domain and glycan cap regions of EboGP, that when anchored to the cell surface is sufficient to antagonize tetherin activity. Conversely, these data indicate that the mucin domain and the extracellular region of the GP_2_ subunit of EboGP are dispensable for anti-tetherin activity.

### 3.2. Chimeras Reveal a Specific Requirement for the EboGP Membrane Spanning Domain (msd)

To differentiate whether the activity of sGP-TM_(GP)_ was due to the physical anchoring of EboGP N-terminal region to the membrane or if there was a specific requirement for the Ebola virus msd, chimeras were constructed with heterologous membrane anchoring domains from other type I membrane proteins appended to the C-terminus of sGP (Figure 1a,b). The msd from the avian glycoprotein TVA and from human ACE2 were appended to sGP creating sGP-TM_(TVA)_ and sGP-TM_(ACE2)_ respectively. The expression of these chimeras was analyzed by flow cytometry (Figure 1e) and immunoblot analysis (Supplementary Figures S2 and S3) and all of the chimeras were expressed, albeit at varying levels. One construct, sGP-TM_(TVA)_, seemed to express poorly in the cell lysates when assessed by immunoblot (Supplementary Figure S2). However, flow cytometry analysis shows that sGP-TM_(TVA)_ is well expressed on the cell surface (Figure 1e). The ability of the chimeras to promote virion release was assessed using an Ebola VLP budding assay as described above. In contrast to the results with the Ebola msd, sGP-TM_(ACE2)_ and sGP-TM_(TVA)_ were unable to antagonize tetherin activity as judged by their inability to significantly promote particle release even at the highest level of expression (Figure 1c,d).

Both full-length Ebola glycoprotein and tetherin have been reported to localize to glycolipid-enriched or lipid raft regions of the membrane [7,34,35]. We hypothesized that the msd and short cytosolic domain of EboGP might provide lipid raft localization to the sGP-TM_(GP)_ chimera thus facilitating tetherin antagonism. To address this theory, sequences for an alternatively spliced form of avian TVA that encodes a glycosylphosphatidylinositol (GPI)-linked anchor [36,37] were appended onto sGP to produce sGP-GPI_(TVA)_ (Figure 1a,b). Western blot and flow cytometry analysis demonstrated that this chimera was expressed and routed to the cell surface (Figure 1e, Supplementary Figure S3). Expression of this chimera with Ebola VP40 and tetherin demonstrated that it is unable to promote virion release in the presence of tetherin (Figure 1d). In sum, these results demonstrate that fusion of 39 residues containing the msd from the GP_2_ subunit of EboGP onto 320 residues of the ectodomain is sufficient to effectively antagonize human tetherin. Moreover, they reveal a specific requirement for the 39 residues of the EboGP msd.

A notable feature of the Ebola msd is the presence of cysteine residues near the inner membrane surface. In EboGP these two cysteine residues are reported to be acylated, however the functional consequences of acylation remain unknown [38]. To determine if these residues contributed to the observed anti-tetherin function of GP, we replaced amino acids C670 and C672 with alanine residues both individually and in tandem. Similar to other reports, we found that replacing either or both cysteines with alanine residues did not alter the ability of EboGP to release tethered VLPs in a budding assay (Supplementary Figure S4) [26,30].

### 3.3. The Ebola msd Requirement is not Specific for Filoviral Budding

**Figure 2:**
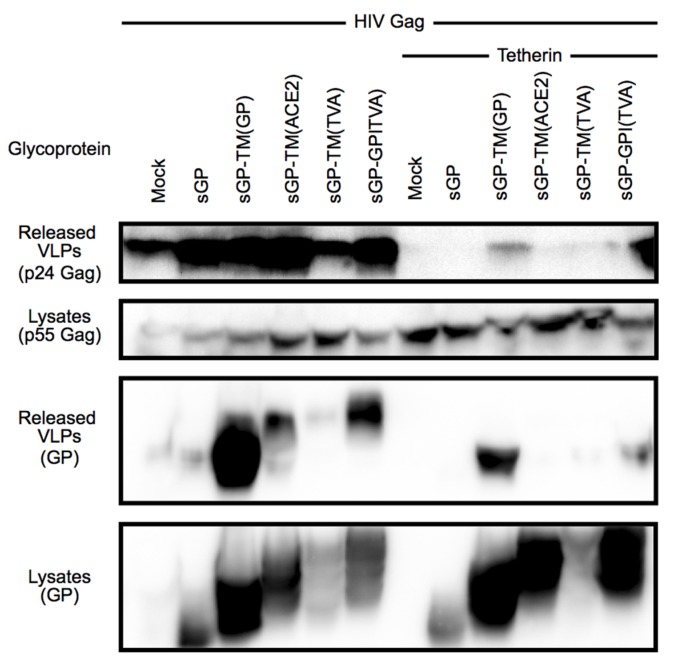
Chimeric sGP glycoproteins incorporate into HIV-1 VLPs but do not promote release of tetherin restricted particles. *Top Panel*: Released HIV-1 VLPs were analyzed by SDS-PAGE and detected by immunoblot for HIV-1 Gag (p24) in supernatant of 293T cells transfected with an HIV-1 Gag encoding vector plus chimeric sGPs with or without tetherin; *Second Panel*: Expression of HIV Gag. Cell lysates of the transfected cells were analyzed by immunoblot for expression of HIV Gag (p55); *Third Panel*: Incorporation of GPs into VLPs. The immunoblot in the top panel was stripped and probed with a polyclonal antibody against EboGP; *Fourth Panel*: Expression of sGP and chimeric sGPs. The immunoblot from the second panel was stripped and expression of GP was analyzed in the cell lysates. These results are representative of two independent experiments using PEI instead of Lipofectamine 2000.

To address whether the requirement for the EboGP msd is specific for budding of Ebola VP40 or if the EboGP msd is able to promote release of other viral particles that are restricted by Tetherin, we tested several of the EboGP chimeras for their effect upon HIV-1 VLP budding in the presence of human tetherin. Similar to the results with VP40, the sGP-TM_(GP)_ chimera allowed budding of HIV particles from tetherin expressing cells (Figure 2, top panel). As was the case for filamentous Ebola VP40 particles, neither the ACE2 or TVA heterologous membrane spanning regions, nor the TVA GPI anchor could substitute for the msd of EboGP to promote efficient HIV-1 budding (Figure 2, top panel). Although the sGP-TM_(TVA)_ chimera was poorly expressed in these experiments, which might account for its inability to counteract tetherin, both sGP-TM_(ACE2)_ and sGP-GPI_(TVA)_ were well expressed (Figure 2, fourth panel) yet unable to promote HIV-1 particle budding.

One possible reason for the failure of these chimeras to relieve tetherin restriction could be that they are unable to localize to the particle budding sites. To address this hypothesis the incorporation of EboGP msd chimeras into HIV-1 particles in the absence of tetherin was analyzed. As can be seen in Figure 2 (third panel, left hand lanes), sGP-TM_(GP)_, sGP-TM_(ACE2)_ and sGP-GPI_(TVA)_ were effectively incorporated into HIV-1 particles while sGP-TM_(TVA)_ was poorly expressed and incorporated. This finding demonstrates that these chimeric glycoproteins, which are unable to relieve tetherin restriction, are not excluded from sites where HIV-1 viral particles bud and suggests that an ability to move the EboGP ectodomain into the site of budding is not sufficient to relieve tetherin restriction. While the exact nature of the features critical for release of tetherin restricted virions within the Ebola virus msd remain to be elucidated, overall, these chimera studies point to a critical role of the EboGP msd for function as a tetherin antagonist. Moreover, they identify the amino terminal 295 residues of sGP, when appended to the EboGP msd, as sufficient for tetherin antagonism.

### 3.4. The Tetherin Amino-Terminal Region is not Required for EboGP Recognition

**Figure 3:**
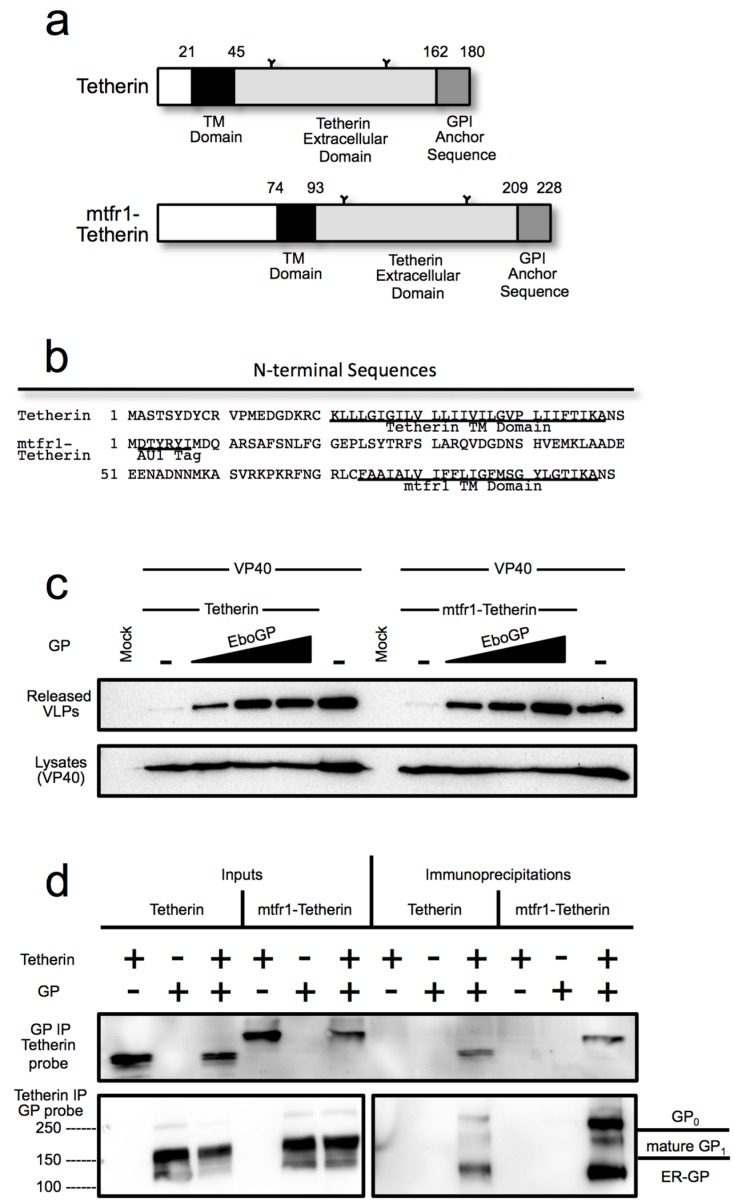
(**a**) Schematic diagram of human tetherin and the chimeric mtfr1-tetherin containing a cytoplasmic tail and transmembrane domain from mouse transferrin receptor 1 (mtfr1); (**b**) A comparison of the N-terminal amino acid sequence of tetherin and mtfr1-tetherin; (**c**) A budding assay comparing the ability of the Ebola virus glycoprotein to release VLPs retained by tetherin or mtfr1-tetherin. 293T cells were transfected with VP40-FLAG and increasing amounts of EboGP with either human tetherin or chimeric mtfr-tetherin as indicated. *Top Panel*: Purified VLPs were analyzed by SDS-PAGE/immunoblot using an anti-FLAG antibody for detection. *Bottom Panel*: 293T cell lysates were analyzed by SDS_PAGE/immunoblot, and probed with the anti-FLAG antibody to confirm VP40 expression; (**d**) Co-immunoprecipitation analysis comparing the ability of tetherin and mtfr1-tetherin to interact with EboGP. Lysates from 293T cells expressing tetherin and/or EboGP were analyzed by SDS-PAGE/immunoblot either directly (Inputs) or after immunoprecipitation with antibody specific for GP (top panel) or tetherin (bottom panel). Molecular mass is shown to the left in kD. The major forms of EboGP are indicated to the right. The left middle panel is a lower exposure of the right middle panel to visualize the overexposed lanes.

A specific requirement for the EboGP msd might suggest direct recognition of the tetherin msd by EboGP. To test this hypothesis, the tetherin msd and N-terminal cytoplasmic domains were replaced with the domains from mouse transferrin receptor protein 1 to generate a chimeric protein, mtfr1-tetherin (Figure 3a,b). Previous studies with similar chimeras have shown that the specific msd of tetherin is dispensable for tetherin function [27,39]. To confirm that mtfr1-tetherin retained the ability restrict virion release, a VLP budding assay was employed (Supplementary Figure S5). The chimera was able to block VLP release, although the activity was slightly reduced based on expression level compared to wild-type (wt) tetherin. Having demonstrated that this chimeric tetherin is active, the ability of EboGP to counteract the activity of mtfr1-tetherin was assessed using a VLP budding assay. As seen in Figure 3c, EboGP was able to promote VLP release similarly from cells expressing wt tetherin or mtfr1-tetherin. This result suggests that EboGP does not require the tetherin msd in order to recognize tetherin and impair its activity.

Previously, EboGP and tetherin have been shown to interact by co-immunoprecipitation (IP) [2]. To determine whether EboGP physically interacts with the chimeric mtfr1-tetherin, wt tetherin or the mtfr1-chimera were co-expressed with EboGP in 293T cells. Cell lysates were immunoprecipitated with a polyclonal anti-EboGP antibody followed by western blot analysis for tetherin. As seen in Figure 3d, EboGP effectively immunoprecipitates both wt and the chimeric tetherin proteins. To verify the interaction, the reciprocal IP was performed using an antibody that reacts with the ectodomain of tetherin to precipitate, followed by western analysis for EboGP (Figure 3d). As was previously noted [2], it appears that the immature forms of EboGP preferentially interact with tetherin—and here this finding is seen for both wild type and the chimeric mtfr1-tetherin. These experiments demonstrate that while the msd domain of EboGP is required for tetherin antagonism, recognition does not require specific intra-membrane sequences in tetherin.

### 3.5. The Glycan Cap of EboGP is Required to Antagonize Tetherin

**Figure 4:**
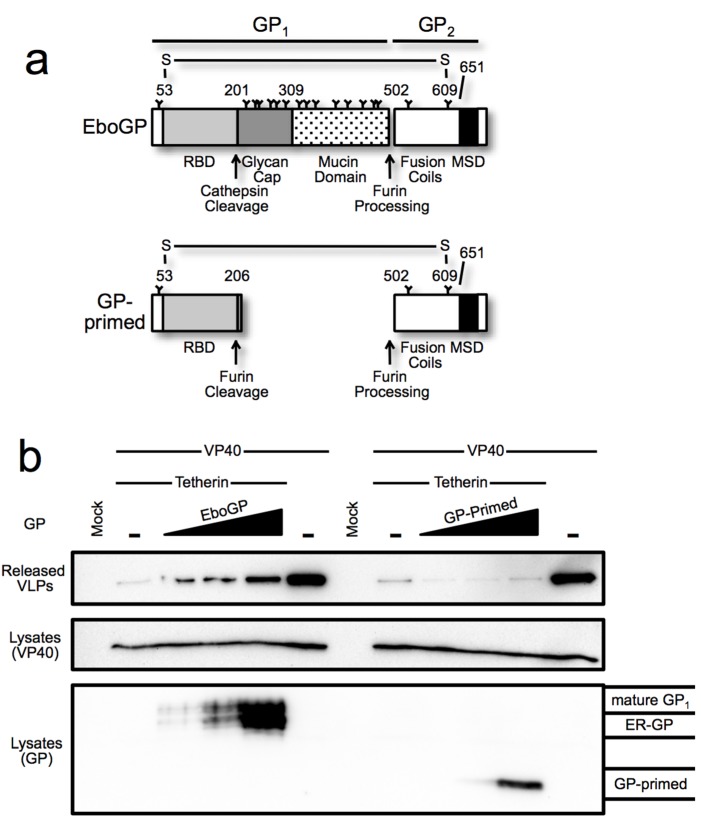

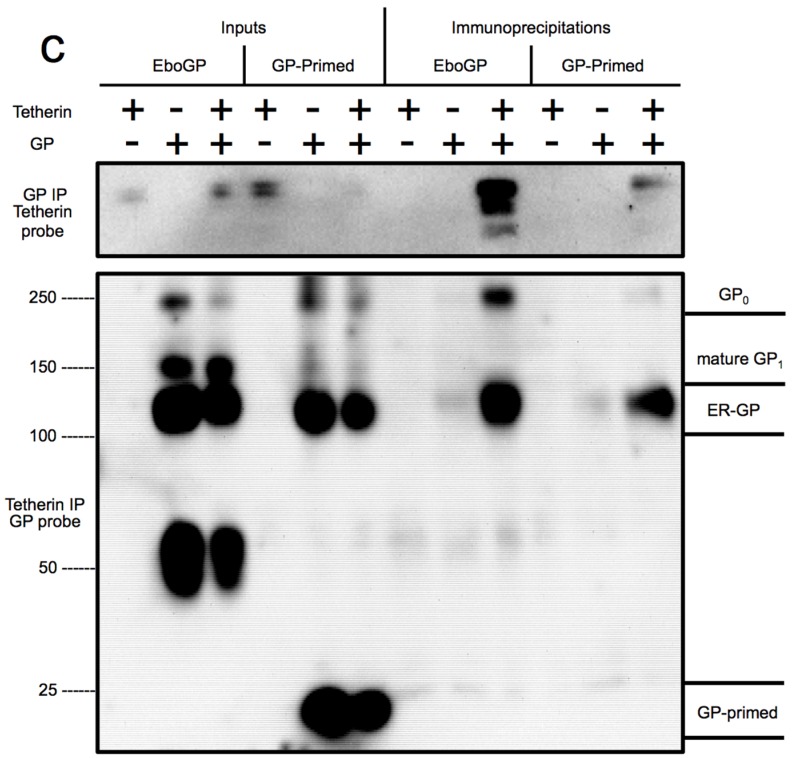
(**a**) Schematic diagram depicting the domains of EboGP and GP-primed, a construct that mimics the glycoprotein produced during Ebola virus entry by introduction of a furin cleavage site at the position where cathepsin processing normally occurs; (**b**) Ebola VLP budding assay comparing GP-primed to EboGP. *Top Panel*: Purified supernatants of 293T cells transfected with the indicated expression plasmids were analyzed by SDS-PAGE and probed with anti-FLAG antibody to detect FLAG tagged VP40. *Middle Panel*: 293T cells producing the VLPs were lysed in TritonX-100 buffer, analyzed by SDS-PAGE and immunoblot using α-FLAG antibody. *Bottom Panel*: The blot from the middle panel was stripped and re-probed with polyclonal antibody against GP to detect the differential forms of the filovirus glycoproteins; (**c**) EboGP and GP-primed interactions with tetherin. Analysis of cell lysates (input) or immunoprecipitated proteins from 293T cells transfected with tetherin and EboGP or GP-primed as indicated. *Top Panel*: Precipitation using polyclonal α-EboGP sera flowed by SDS-PAGE and detection with an *α*-tetherin monoclonal antibody. *Bottom Panel*: A reciprocal analysis, using an α-tetherin antibody for immunoprecipitation and detection with *α*-EboGP sera. Molecular mass is shown in kD. Indicated on the right are the immature (ER) form of the Ebola virus glycoprotein, GP_0_ and the mature forms of GP-primed and EboGP.

Among filoviruses, the glycan cap is a moderately conserved (~55% identity) glycosylated domain within the viral glycoprotein GP_1_ subunit. This region is proteolytically cleaved by cellular cathepsins during filoviral entry to reveal a binding site for NPC1, the conserved receptor [22,40,41]. Aside from occluding the receptor-binding site, no other function has been ascribed to the glycan cap. Indeed, virus produced in which the cap domain is removed by *in vitro* proteolysis is more infectious than wt virions, demonstrating that this domain is dispensable for the cell entry function of EboGP [33,42,43]. To examine the role of this domain in antagonizing tetherin, an EboGP mutant was constructed such that sequences encoding the glycan cap could be readily removed [33]. This was accomplished by inserting a consensus furin cleavage site at the point in a disordered loop where cathepsin cleavage usually occurs generating GP-primed (Figure 4a). The added furin site allows GP-primed to be cleaved by host proteases during production of the glycoprotein, thus mimicking the cleavage produced by cathepsins during entry [33]. To determine the role of the glycan cap in promoting virion release, a VLP budding assay was used to compare EboGP and GP-primed. As seen in Figure 4b, GP-primed did not promote release VLPs even at the highest levels of expression tested.

To ascertain whether the glycan cap domain participates in the EboGP–tetherin interaction, we compared the ability of EboGP and GP-primed to immunoprecipitate tetherin. EboGP and tetherin were co-expressed in 293T cells and the interaction was assessed by co-IP with antibodies to EboGP. The interaction was also verified by performing the reciprocal IP. Both the inputs and immunoprecipitated protein were analyzed by Western blot (Figure 4c). We were able to confirm the EboGP-tetherin interaction, as shown in previous work [2]. Surprisingly, in contrast to the VLP release data, IP of cells expressing GP-primed effectively co-precipitated tetherin. However, an IP with a tetherin antibody precipitated only pre-processed immature GP and not the cleaved form lacking the glycan cap or the mature full-length glycoprotein. Thus, while the glycan cap seems to be important for the anti-tetherin activity of EboGP, it remains unclear whether or not the glycan cap has a role in mediating the tetherin interaction.

## 4. Discussion

Tetherin represents an important barrier to replication of a number of enveloped viruses; consequently viruses have evolved a variety of specific tetherin antagonists. The Ebola virus envelope glycoproteins are effective tetherin antagonists [1,2,12] and have been shown to promote viral spread in tetherin expressing cells [29]. Here we dissect the requirements within the Ebola glycoproteins that are important to counteract tetherin activity. Overall, we find that regions in both the ectodomain and membrane spanning domain of the Ebola virus glycoprotein are necessary and, when expressed as a chimeric protein, sufficient to antagonize tetherin activity.

Analysis of chimeric GP envelope proteins in which the membrane-spanning region of EboGP is replaced by heterologous sequences indicates that this region of the GP_2_ subunit is required for tetherin antagonism. Our findings are similar to recent studies [30] where it was found that the membrane-spanning domain from an arenavirus glycoprotein was unable to replace the EboGP msd. In other studies, it was suggested from co-IP analysis that the GP_2_ subunit is sufficient for an interaction with tetherin [29]. In contrast, our analysis demonstrates that the ability of EboGP to counteract tetherin requires sequences from the GP_1_ subunit, but not from the extracellular sequences within the GP_2_ subunit. This discrepancy likely reflects the different assays used in the analysis or may suggest that the interaction measured by IP is not a surrogate for anti-tetherin activity.

HIV-1 Vpu utilizes sequences within the membrane spanning domain to directly interact with human tetherin [44,45,46]. Indeed this sequence specificity determines the restricted host range of Vpu [47,48]. Given that EboGP also requires the membrane-spanning region of GP_2_ it is tempting to speculate that the Ebola virus glycoprotein also directly recognizes tetherin via the membrane spanning sequences. However, data presented here and elsewhere argue against this hypothesis. First, EboGP recognizes divergent tetherin species where there is low conservation of the tetherin membrane spanning sequence. For example, mouse tetherin has only 38% identity with human, yet it is still effectively antagonized by EboGP [2]. Similarly, data presented here shows that replacing the tetherin membrane spanning region and cytoplasmic tail with mouse transferrin receptor sequences still allows tetherin antagonism. These results are similar to data from Lopez *et al.* analyzing chimeric tetherin proteins [27]. Overall these data suggest that if the EboGP membrane-spanning region recognizes tetherin, it likely does so in a sequence independent manner.

The ability of sGP-TM_(GP)_ to promote virus release suggests that recognition of tetherin requires the amino terminal 320 residues of the Ebola envelope surface glycoprotein but not the extracellular sequences from the GP_2_ subunit. Although the structure of sGP is not determined, by comparison with the crystal structure of full length Ebola GP, sGP-TM_(GP)_ includes the receptor binding and glycan cap domains of GP_1_ [22]. Removal of the glycan cap region of EboGP in the GP-primed mutant by incorporation of a furin protease cleavage site at position 206, abrogates EboGP tetherin antagonist activity. This mutant glycoprotein is analogous to the cathepsin-processed form of EboGP that is fully functional for entry into host cells [23,33]. The inability of GP-primed to affect tetherin activity might suggest that the glycan cap region may have another role in addition to occluding the receptor binding domain—namely tetherin antagonism. Exactly how this region recognizes tetherin, or if it can directly promote Ebola GP interaction with tetherin, remains to be determined. Finally, the differential anti-tetherin activities of the sGP-TM_(GP)_ chimera and GP-primed mutant demonstrate that the tetherin antagonist function can be separated from a role in viral entry.

Ebola viral particles are believed to bud from cholesterol-rich lipid rafts where both the viral matrix and glycoproteins localize [49]. The GPI anchor of tetherin is required for antiviral function [1] and likely acts at least in part by directing the protein to sites of budding [7]. However, anchoring sGP via a GPI tail did not confer anti-tetherin function. Thus localizing sGP at the site of viral budding and tetherin activity does not appear to be sufficient to antagonize tetherin reinforcing our finding that the membrane spanning region of EboGP plays a critical role. Interestingly, although HIV-1 Vpu also localizes to lipid rafts, raft-association is not required to antagonize tetherin activity and promote HIV-1 release [26,50]. This supports the notion that features within the EboGP msd other than lipid raft association are important for antagonism, however the nature of these features or the mechanism by which they act remain obscure.

For the HIV-2 and SIV envelope proteins, extracellular determinants have also been shown to govern tetherin specificity, however with EboGP, the exact nature of the extracellular region needed remains unclear. Additionally, for SIV and HIV-2 envelope proteins, antagonism requires recognition of tetherin through the ectodomain and a highly conserved endocytosis motif in the cytoplasmic tail [51]. In contrast, the short four residue cytoplasmic tail of EboGP has no similar motif, thus GP directed endocytosis is not a likely role for the Ebola msd in the sGP-TM_(GP)_ chimera. Moreover, SIV and HIV-2 envelope proteins appear to restrict tetherin to the trans Golgi network [12,51] whereas no such relocalization has been seen for EboGP [27,29]. Overall the precise mechanism by which these various viral glycoproteins act upon tetherin is obscure. However, our studies localize anti-tetherin activity to 320 ectodomain residues plus 39 amino acids of the EboGP msd. The Ebola viral glycoprotein and the chimeras and mutants we describe provide a platform for addressing these mechanistic questions.

## Supplementary Materials

Figure S1: MSD and N-terminal Sequences; Figure S2: GP expression in lysates 1; Figure S3: GP expression in lysates 2; Figure S4: C-terminal cysteines budding assay; Figure S5: mtfr1-tetherin budding assay.
