# Supplementary material for: Requirements within the Ebola Viral Glycoprotein for Tetherin Antagonism
Source: Viruses. 2015 Oct 26;7(10):5587–602. doi: 10.3390/v7102888 (PMC4632396; doi:10.3390/v7102888)
Supplement: Supplementary file 1 [file viruses-07-02888-s001.pdf]

MSD and N-terminal Sequences

```
EboGP      Q  G  D  N  D  N  W   W  T  G  W  R  Q  W   I  P  A  G  I  G   V  T  G  V  V  I  A   V  I  A  L  F  C  I   C  K  F  V  F  P
      1912 CAGGGGACAATGACAATTG GTGGACAGGATGGAGACAAT GGATACCGGCAGGTATTGGA GTTACAGGCGTTGTAATTGC AGTTATCGCTTTATTCTGTA TATGCAAATTTGTCTTTCCG
                Ectodomain                                                EboGP Transmembrane Domain                     Cytoplasmic Tail
           R  F  E  G  K  P  I   P  N  P  L  L  G  L   D  S  T  R  T  G   H  H  H  H  H  H  *
      2032 CGGTTCGAAGGTAAGCCTAT CCCTAACCCTCTCCTCGGTC TCGATTCTACGCGTACCGGT CATCATCACCATCACCATTG A
                                      V5-His tag
sGP        K  T  S  V  V  R  V   R  R  E  L  L  P  T   Q  G  P  T  Q  Q   L  K  T  T  K  S  W   L  Q  K  I  P  L  Q   W  F  K  C  T  V
       946 AAAACATCAGTGGTCAGAGT CCGGCGCGAACTTCTTCCGA CCCAGGGACCAACACAACAA CTGAAGACCACAAAATCATG GCTTCAGAAAATTCCTCTGC AATGGTTCAAGTGCACAGTC
              sGP     Furin Cut Site                                                            Delta Peptide
           K  E  G  K  L  Q  C   R  I  *
      1066 AAGGAAGGGAAGCTGCAGTG TCGCATCTAA

sGP-TM     K  T  S  V  V  S  R   Q  G  D  N  D  N  W   W  T  G  W  R  Q   W  I  P  A  G  I  G   V  T  G  V  V  I  A   V  I  A  L  F  C
(GP)   946 AAAACATCAGTGGTCTCTAG ACAGGGGGACAATGACAATT GGTGGACAGGATGGAGACAA TGGATACCGGCAGGTATTGG AGTTACAGGCGTTGTAATTG CAGTTATCGCTTTATTCTGT
              sGP        XbaI          EboGP Ectodomain                                      EboGP Transmembrane Domain
           I  C  K  F  V  F  *
      1066 ATATGCAAATTTGTCTTTTA G
             EboGP Cytoplasmic Tail
sGP-TM     K  T  S  V  V  S  R   Q  P  T  L  G  P  P   N  Q  P  P  V  S   I  W  L  I  V  F  G   V  V  M  G  V  I  V   V  G  I  V  I  L
(ACE2) 946 AAAACATCAGTGGTCTCTAG ACAGCCAACACTTGGACCTC CTAACCAGCCCCCTGTTTCC ATATGGCTGATTGTTTTTGG AGTTGTGATGGGAGTGATAG TGGTTGGCATTGTCATCCTG
              sGP        XbaI            ACE2 Ectodomain                                     ACE2 Transmembrane Domain
           I  F  T  G  I  R  D   R  K  K  K  N  K  A   R  S  G  E  N  P   Y  A  S  I  D  I  S   K  G  E  N  N  P  G   F  Q  N  T  D  D
      1066 ATCTTCACTGGGATCAGAGA TCGGAAGAAGAAAAATAAAG CAAGAAGTGGAGAAAATCCT TATGCCTCCATCGATATTAG CAAAGGAGAAAATAATCCAG GATTCCAAAACACTGATGAT
                                                  ACE2 Cytoplasmic Tail
           V  Q  T  S  F  *
      1186 GTTCAGACCTCCTTTTAG

sGP-TM     K  T  S  V  V  L  E   P  T  D  N  G  T  E   A  P  T  V  P  A   P  G  R  A  L  P  A   R  N  H  G  R  M  W   M  L  I  T  A  V
(TVA)  946 AAAACATCAGTGGTCCTCGA GCCCACGGACAACGGCACAG AGGCTCCCACTGTCCCTGC TCCTGGACGTGCTCTGCCAGC CAGGAATCACGGCCGCATGT GGATGCTGATCACTGCAGTG
              sGP        XhoI                             TVA Ectodomain
           L  L  C  C  L  V  A   V  G  G  I  A  A  W   G  K  S  K  A  K   S  R  S  D  I  F  S   L  E  S  A  S  K  E   L  L  V  P  D  K
      1066 CTCCTGTGCTGCCTGGTAGC TGTGGGTGGTATCGCTGCAT GGGGGAAGTCCAAAGCAAA AAGCAGGTCTGACATCTTCAG TCTTGAAAGCGCATCCAAGG AGCTGCTGGTGCCTGACAAG
              TVA Transmembrane Domain                                                          TVA Cytoplasmic Tail
           S  Q  A  D  L  F  S   *
      1186 AGCCAGGCAGACTTGTTCTC CTGA

sGP-GPI    K  T  S  V  V  S  R   P  T  D  N  G  T  E   A  P  T  V  P  A   P  G  R  A  L  P  A   R  N  H  G  R  M  W   M  L  I  T  A  G
(TVA)  946 AAAACATCAGTGGTCTCTAG ACCCACGGACAACGGCACAG AGGCTCCCACTGTCCCTGC TCCTGGACGTGCTCTGCCAGC CAGGAATCACGGCCGCATGT GGATGCTGATCACTGCAGGG
              sGP        XbaI                                               TVA Ectodomain and GPI anchor signal sequence
           I  F  C  C  E  L  V   R  W  D  *
      1186 ATCTTTTGCTGTGAGCTGGT GAGATGGGACTGA

Tetherin   M  A  S  T  S  Y  D   Y  C  R  V  P  M  E   D  G  D  K  R  C   K  L  L  L  G  I  G   I  L  V  L  L  I  I   V  I  L  G  V  P
         1 ATGGCATCTACTTCGTATGA CTATTGCAGAGTGCCCATGG AAGACGGGGATAAGCGCTGT AAGCTTCTGCTGGGGATAGG AATTCTGGTGCTCCTGATCA TCGTGATTCTGGGGGTGCCC
                 Tetherin Cytoplasmic Tail                                            Tetherin Transmembrane Domain
           L  I  I  F  T  I  K   A  N  S
       121 TTGATTATCTTCACCATCAA GGCCAACAGC

mtfr1-     M  D  T  Y  R  Y  I   M  D  Q  A  R  S  A   F  S  N  L  F  G   G  E  P  L  S  Y  T   R  F  S  L  A  R  Q   V  D  G  D  N  S
Tetherin 1 ATGGATACATATCGATACAT TATGGATCAAGCCAGATCAG CATTCTCTAACTTGTTTGGT GGGGAACCATTGTCATACAC CCGGTTTAGCCTTGCTCGGC AAGTAGATGGAGATAACAGT
              AU1 tag                                                   mtfr1 Cytoplasmic Tail
           H  V  E  M  K  L  A   A  D  E  E  E  N  A   D  N  N  M  K  A   S  V  R  K  P  K  R   F  N  G  R  L  C  F   A  A  I  A  L  V
       121 CATGTGGAGATGAAACTGGC TGCAGATGAAGAAGAAAATG CCGACAATAACATGAAGGCT AGTGTCAGAAAACCCAAGAG GTTTAATGGAAGACTCTGCT TTGCAGCTATTGCACTAGTC

           I  F  F  L  I  G  F   M  S  G  Y  L  G  T   I  K  A  N  S
       241 ATTTTCTTCTTGATTGGATT CATGAGTGGCTACCTGGGCA CCATCAAGGCCAACAGC
                 mtfr1 Transmembrane Domain
```

Supplemental Figure 1. A nucleotide sequence list comparing the unique C-terminal or N-terminal domains for each construct used in this study. The amino acid translation is given above each sequence and annotations are indicated below each sequence.

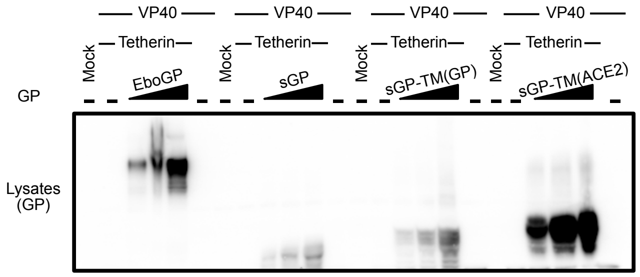

Supplemental Figure 2. SDS-PAGE immunoblot of 293T cell lysates analyzing expression of the glycoproteins used in the budding assay from Figure 1C. The immunoblot of the cell lysates from Figure 1C was stripped and reprobed with the R12 antibody to detect verify expression of the constructs in the budding assay.

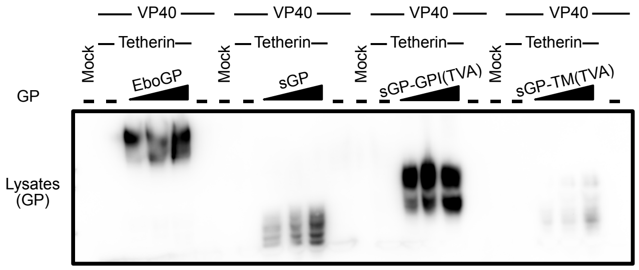

Supplemental Figure 3. Immunoblot analyzing glycoprotein expression in the 293T cell lysates from the budding assay in Figure 1D. The western blot from the budding assay in Figure 1D was stripped and re-probed with the R12 antibody to confirm expression of the new constructs.

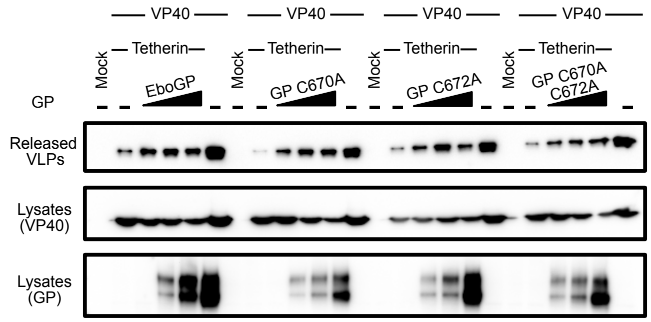

Supplemental Figure 4. A VP40 VLP budding assay assessing the ability of EboGP to antagonize tetherin with either one or both membrane proximal cysteines modified to an alanine. *Top Panel*: An immunoblot depicting purified VLPs released by each of the glycoproteins, suggesting that modification of the cysteine residues at 670 and 672 do not affect VLP release. *Middle Panel*: Cellular lysates were also analyzed by immunblot to verify the expression of VP40. *Bottom Panel*: The middle panel immunoblot was stripped and reprobed with the R12 antibody to detect glycoprotein expression in the cellular lysates.

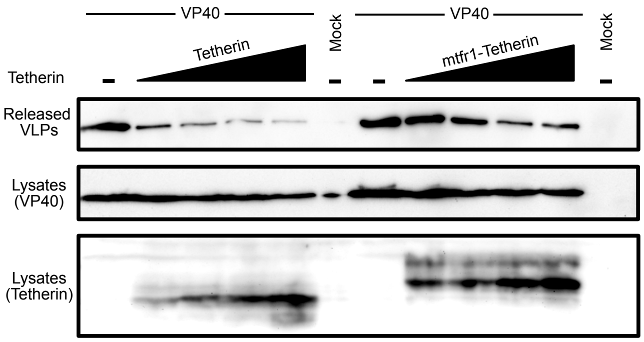

Supplemental Figure 5. A VP40 VLP budding assay comparing the ability of tetherin and mtfr1-tetherin to prevent release of VLPs into the supernatant. *Top Panel*: VLPs analyzed by immunoblot showing the effectiveness of both tetherin and mtfr1-tetherin in retaining budded particles. *Middle Panel*: An immunoblot showing the expression of VP40 in the corresponding cellular lysates. *Bottom Panel*: The immunoblot from the middle panel was reprobed for tetherin expression.
